# Sediment Sampling in Estuarine Mudflats with an Aerial-Ground Robotic Team

**DOI:** 10.3390/s16091461

**Published:** 2016-09-09

**Authors:** Pedro Deusdado, Magno Guedes, André Silva, Francisco Marques, Eduardo Pinto, Paulo Rodrigues, André Lourenço, Ricardo Mendonça, Pedro Santana, José Corisco, Susana Marta Almeida, Luís Portugal, Raquel Caldeira, José Barata, Luis Flores

**Affiliations:** 1 INTROSYS SA, Introsys—Global Control System Designers, Parkim- Parque Industrial da Moita Rua dos Girassóis, n°1, Lote 6, Armazém A3, 2860-274 Moita, Portugal; magno.guedes@introsys.eu (M.G.); andre.silva@introsys.eu (A.S.); raquel.caldeira@introsys.eu (R.C.); luis.flores@introsys.eu (L.F.); 2 CTS-UNINOVA, Universidade Nova de Lisboa (UNL), FCT Campus, 2829-516 Caparica, Portugal; fam@uninova.pt (F.M.); emp@uninova.pt (E.P.); paulomrpt@gmail.com (P.R.); andre.lourenco@uninova.pt (A.L.); rmm@uninova.pt (R.M.); jab@uninova.pt (J.B.); 3 ISCTE-Instituto Universitário de Lisboa (ISCTE-IUL), 1649-026 Lisboa, Portugal; pedro.santana@iscte.pt; 4 Instituto de Telecomunicações (IT), 1049-001 Lisboa, Portugal; 5 Centro de Ciências e Tecnologias Nucleares (C2TN), Instituto Superior Técnico, Universidade de Lisboa, E.N. 10 km 139.7, 2695-066 Bobadela LRS, Portugal; corisco@ctn.tecnico.ulisboa.pt (J.C.); smarta@ctn.tecnico.ulisboa.pt (S.M.A.); 6 Agência Portuguesa do Ambiente (APA), Agência Portuguesa do Ambiente, I.P., Rua da Murgueira, 9/9A-Zambujal, Ap. 7585, 2610-124 Amadora, Portugal; luis.portugal@apambiente.pt

**Keywords:** multi-robot system, field robots, UGV, UAV, environmental monitoring, radiological monitoring, heavy metals monitoring, estuarine mudflats

## Abstract

This paper presents a robotic team suited for bottom sediment sampling and retrieval in mudflats, targeting environmental monitoring tasks. The robotic team encompasses a four-wheel-steering ground vehicle, equipped with a drilling tool designed to be able to retain wet soil, and a multi-rotor aerial vehicle for dynamic aerial imagery acquisition. On-demand aerial imagery, properly fused on an aerial mosaic, is used by remote human operators for specifying the robotic mission and supervising its execution. This is crucial for the success of an environmental monitoring study, as often it depends on human expertise to ensure the statistical significance and accuracy of the sampling procedures. Although the literature is rich on environmental monitoring sampling procedures, in mudflats, there is a gap as regards including robotic elements. This paper closes this gap by also proposing a preliminary experimental protocol tailored to exploit the capabilities offered by the robotic system. Field trials in the south bank of the river Tagus’ estuary show the ability of the robotic system to successfully extract and transport bottom sediment samples for offline analysis. The results also show the efficiency of the extraction and the benefits when compared to (conventional) human-based sampling.

## 1. Introduction

Bottom sediments in estuarine bays once stressed by effluent discharges from industries and urban environments were exposed to the deposition of contaminants by physical settling of particulate matter and chemical sorption from water. The process is ruled by physical conditions, such as water turbulence, contact time and sediment surface topography, and by the chemical nature and physical form of contaminants in the water column. All of these variables tend to be spatially complex, leading to heterogeneous distribution patterns of contaminants. In time, the hydrodynamics-induced redistribution of particles will intensify the spatial heterogeneity created by a primary deposition. The presence of inorganic toxicants, like radionuclides and heavy metals, in the mudflats of major rivers is an issue of public concern and environmental relevance, pressing the need for an extensive survey of the intertidal mudflat. Continuous dredging of navigation channels of river estuaries and human scavenging activities, such as intensive clam harvesting, are likely to promote the re-suspension of both surface and anoxic bottom sediments, causing the remobilization of adsorbed toxicants [1].

Currently, environmental monitoring surveys in estuarine mudflats are performed by human experts handling manual sampling tools, such as the manual auger lanyards for extracting cylindrical columns (also known as cores) of soil or sediment to a certain depth. To obtain cores in continental areas, these devices are often endowed with an auxiliary motor. With these manual tools, sediment samples are collected and then carried from the site to the lab for an offline analysis. Walking in the mudflat, handling the sampling tools, transporting the samples and ensuring that these are properly tagged and geo-referenced are just a few of several physically-demanding, costly and time-consuming challenges humans must face in these sampling campaigns. To mitigate these difficulties, this paper presents a robotic team developed to support sampling operations in mudflats and, as a result, to facilitate thorough spatio-temporal sampling campaigns therein.

**Figure 1:**
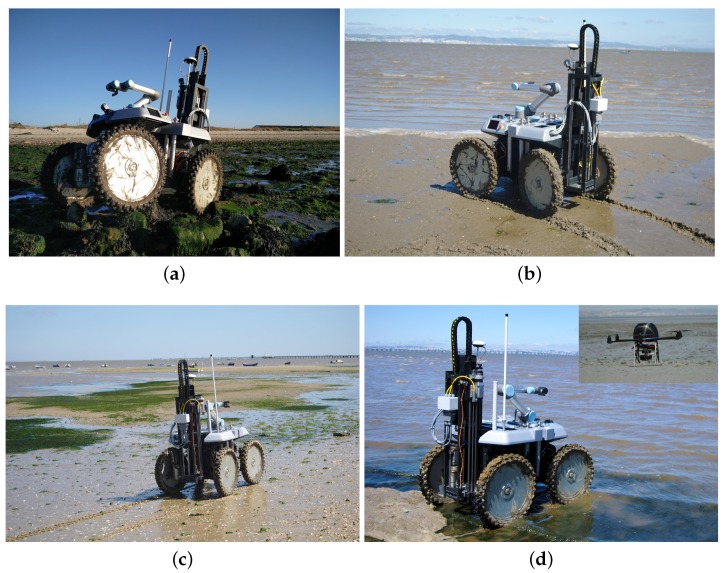
The robotic team in the field trials. The ground vehicle is retrofitted with the drilling tool, which is recharged by a six-DOF arm. The aerial vehicle is equipped with a high resolution camera whose pose is controlled with an active gimbal. (**a**) The ground vehicle over irregular terrain. A passive longitudinal axle allows the robot to follow the terrain’s unevenness. (**b**) The ground vehicle traveling over muddy terrain. (**c**) The ground vehicle over terrain covered with seaweed, small stones and clam shells. (**d**) The ground vehicle traveling over water after three collected samples and the aerial vehicle surveying the field trials area.

Figure 1 depicts the robotic team, which is composed of a wheeled Unmanned Ground Vehicle (UGV) and a multirotor Unmanned Aerial Vehicle (UAV). The UGV is a rugged version of the INTROBOT all-terrain robotic platform [2], whose high clearance to the ground, four-wheel steering capabilities and passive compliance to terrain roughness offer high maneuverability in the mudflat. Via a (de)attachable and electrically-actuated custom drilling tool, the UGV is able to extract cylindrical bottom sediment samples, each with a section of 6 cm and a depth of 45 cm. These samples are obtained by penetrating the soil with a cylindrical container through a mix of linear and rotational movement. These containers are designed to ensure that the wet soil sample does not fall from the container and that different depths do not get mixed, which is key for a proper off-line depth-based sediment analysis. A compliant six-DOF robotic arm attached to the UGV interacts with the drilling tool in order to exchange up to nine cylindrical containers.

The statistical significance and accuracy of environmental monitoring campaigns depend heavily on the execution of well-prepared experimental protocols, which must be adapted to the characteristics of the sampling site by a human expert. To support the human expert in this process, the UAV provides an aerial mosaic from a set of images acquired right before the mission’s onset with a high resolution camera. The UAV is also used by the expert to supervise the ground vehicle’s operation. Estuarine environments are ruled by intense hydrodynamic processes, which cause considerable morphology changes throughout a single day and, thus, rendering satellite imagery rapidly outdated. By relying on a UAV, the human expert gets access to up-to-date imagery.

Current experimental protocols for radiological and heavy metals monitoring in mudflats (e.g., [3]) are based on the assumption that the sampling campaign is performed by humans handling manual tools. If robotic systems are to be accepted by the environmental monitoring community, then these must be somehow included in the experimental protocols already in practice. Towards contributing in this direction, this paper proposes a preliminary experimental protocol that takes into account the inherent capabilities of the robotic system.

To validate the proposed system, a set of field trials was performed in the estuarine intertidal mudflat of the river Tagus (Portugal). Several bottom sediment samples were collected and processed in the lab. These trials showed the ability of the robotic system to maneuver in several types of terrain that can be found in estuarine environments, which includes dry sand, wet sand, ponds and mud. The results also show the ability of the system to drill efficiently in these terrains and, more importantly, that the collected sediment samples are proper for off-line laboratory analysis. The results also show the benefits of the robotic sampling process when compared to (conventional) human-based sampling.

**Figure 2:**
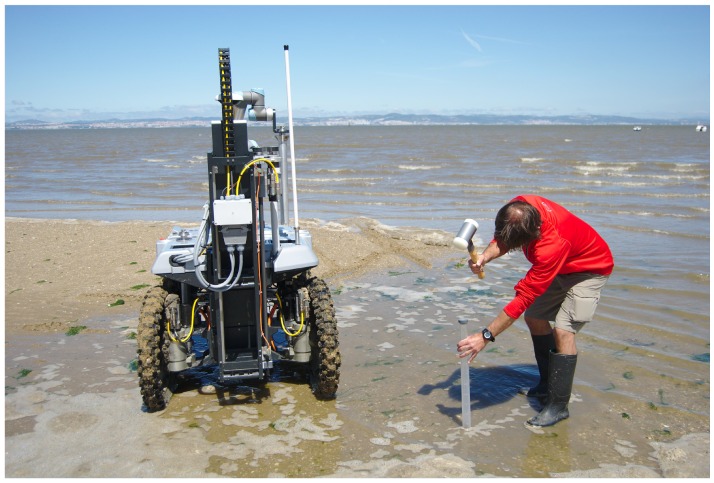
The ground vehicle drilling the terrain side-by-side a human expert handling a conventional manual tool. The human expert hammers an acrylic hollow cylinder until a desired depth is reached. Then, the cylinder must be gently pulled in order to ensure that the sediments do not fall. All of this process is time consuming and physically demanding. Conversely, the robot drills effortlessly and stores up to nine samples before it needs to return to the base.

Figure 2 depicts an arms-race between the robotic system and a human expert collecting sediment samples with a conventional manual tool. As will be shown in Section 6, the human expert managed to perform faster than the robot. However, the expert’s speed varied considerably with the composition of the terrain, whereas the robot kept a homogeneous performance. Moreover, human efficiency drops considerably as the number of samples grows. It should be noted as well that, with the robotic system, all samples are accurately geo-referenced, and there is no need for the human to carry equipment or samples once these are collected.

This paper is an extended and improved version of a conference paper [4], and it is organized as follows. Section 2 surveys related work in environmental monitoring and field robotics. Section 3 presents the devised experimental protocol. Then, Section 4 describes the robotic elements developed to match the requirements specified in the experimental protocol. The way the robotic elements cooperate among themselves and with the human operator is presented in Section 5. Results obtained from a set of field trials are subsequently presented in Section 6. Finally, a set of conclusions and future work avenues are given in Section 7.

## 2. Related Work

Environmental monitoring in remote and vast environments is a highly relevant societal problem that is being addressed by several research communities in many disparate ways. Although originally performed by humans, current approaches tend to exploit technology as much as possible. With technology, environmental monitoring can be faster, more accurate and more significant. With the support of technology, it becomes possible to reduce the physical burden of complex surveys and to focus human effort on the design of experimental protocols, their execution monitoring and subsequent data analysis.

A common approach for technology-driven environmental monitoring is to exploit remote sensing techniques, either based on aerial vehicles or satellites (e.g., [5]). However, being in situ is often required, as many of the environmentally-relevant variables are unavailable otherwise. A common solution for large scale in situ monitoring is to deploy wireless sensor networks in the environment [6,7]. These sensor networks can work as simple data gatherers or as complete remote laboratories. Example applications include the monitoring of volcanoes [8], rivers [9] and underground mines [10]. To allow a better coverage of the environment, sensor networks may encompass mobile robotic elements (e.g., [11]). In this paper, we exploit the fact that, if provided with sufficient storage capacity, mobile robots can also be used for retrieving the collected samples, enabling their off-line laboratory processing.

Although not yet mainstream in environmental monitoring campaigns, field robots are steadily paving their way towards real-life applications. Robots have already been validated as valuable tools for exploring Mars [12], subterranean spaces [13], underwater caves [14], marine environments [15] and riverine environments [16], among others. Robots have also been used for radiological monitoring in emergency-response situations [17]. Please refer to [18,19] for two surveys on the application of robotics to the environmental domain.

To collect sediment samples, robots need to be equipped with sediment sampling tools and store the collected samples. The high diversity of soils that a robot must face in a sampling campaign renders the design of sampling tools a complex problem. For instance, the same tool must be able to penetrate the soil without revolving it and then must be able to pull the sample without dropping or draining it. The following paragraphs overview previous efforts in devising generic soil sampling tools, that is not specifically devised for robotic operation.

Some pneumatic drilling tools have been proposed for operation on unstable soil, including submerged areas, while preserving the structure of the sampled soil [20]. In alternative to pneumatic actuators, some drilling devices have been devised to rely on hydraulic actuators [21,22,23,24,25,26]. To reduce soil resistance while drilling, vibration can be added to the process [27]. Despite the value of all of these early attempts for devising soil sampling tools, relying on hydraulic and pneumatic actuators limits considerably their applicability in mid-sized robotic platforms.

Electrically-driven drilling mechanisms for soil sampling have also been proposed [28,29]. However, these systems use linear actuators to penetrate the soil without including some rotary motion, which is essential to reduce soil resistance while drilling. Although other devices have been designed to include such a rotary motion [30,31], they are unable to prevent the soil getting revolved during the drilling process. This hampers the assessment of contaminants’ vertical distribution in the collected sample.

Complete soil sampling systems that can be towed by a small tractor or other agricultural vehicle have also been proposed [32,33,34,35]. However, these systems have been designed for the extraction of soil only at the surface level, which prevents their use in collecting samples for the purpose of the analysis of soil strata at variable depths.

Instead of employing complex drilling tools, it is also possible to consider the use a robotic arm manipulating small sampling containers [36]. However, such a solution is unable to reach enough sample depth and volume for a significant off-line assessment of the vertical distribution of contaminants. Self-burying robots [37,38] are being proposed as a promising alternative to drilling tools. These robots could virtually reach any depth, providing fine control in the sampling process. However, it is still unclear how these robots can extract significant sample volumes for the purpose of an extensive monitoring campaign.

As an alternative to these previous approaches, we present a complete robotic system for mudflats, equipped with an electrically-driven drilling tool capable of exploiting both linear and rotary motion to reduce soil resistance without revolving the soil. To reduce human intervention, the drilling tool is recharged automatically by a compliant six-DOF robotic arm.

## 3. Experimental Protocol for Robotic Sampling of Estuarine Mudflats

The development of the presented robotic system was driven by a particular environmental monitoring case study: radiological and heavy metals monitoring in the mudflats along a major river’s estuary. The goal was to ensure that the robotic tool was able to cope with a realistic and valuable mission, as defined by environmental monitoring experts. Moreover, this design approach allowed roboticists and environment scientists to co-develop the robots and a preliminary experimental protocol for the case study at hand. This experimental protocol defines the sequence of actions that robots and humans must perform, as well as the sampling procedure that must be executed.

This section starts by describing the case study and its motivation. Subsequently, the mission workflow is presented. Under a pragmatic perspective, this workflow involves the robots and the human experts. Then, the sampling procedure is detailed. Finally, the way the samples should be stored, conserved and processed in the lab is described. With this multi-disciplinary approach, we expect to contribute towards closing the gap between the robotics and the environmental monitoring communities.

### 3.1. Case Study

The specification, development and validation of the robotic system relies on Tagus River’s estuarine bay as the main case study (see Figure 1). The selection of this case study stems from two main observations. First, as will be shown below, studying Tagus’s estuary is a remarkably relevant problem from an environmental monitoring standpoint. Second, Tagus’s estuary is representative of a vast set of estuaries influenced by major littoral cities, such as Lisbon. The muddy sediments of the Tagus River’s estuary have been exposed to decades of contaminant deposition from local industries. The run-off and wind spreading of particulate materials coming from the phosphogypsum stockpile of a disabled phosphate plant, near the city of Barreiro, have been a source for localized enhanced concentrations of natural radioisotopes of the uranium family. The radiological impact of the Barreiro phosphate industry due to uranium 238 descendants, lead 210 and polonium 210, in the bottom sediments and in the water column particulate matter, has been described in [39].

Other industries set on both sides of the estuarine bay contributed to the dispersion of toxic metals, like mercury, cadmium or arsenic, and a variety of other different contaminants, including Polycyclic Aromatic Hydrocarbons (PAH) and organometallic compounds that have been previously reported, suggesting a deterioration of water and sediment quality in some critical areas of the Tagus estuary [39]. The construction of the Vasco da Gama Bridge (roughly 25 km upstream the estuary mouth), from September 1994–December 1998, caused additional disturbance, promoting the remobilization of anoxic contaminated sediments. This fact led to the temporary solubility of toxicant metals followed by re-adsorption to the particulate phase [40].

The early 2000s was a time for some observable changes in both the presence of human activity and the physiognomy of the estuarine mudflat extending through the shoreline from Barreiro to Alcochete. The introduction of the invasive Asian clam *Ruditapes philippinarum* and its massive population expansion triggered an intense activity of clam harvesting for human consumption without any control of toxicants. The scavenging action of hundreds of clam harvesters and boat clam dredging, scattered over a wide area of shallow and low depth water of the estuarine bay, enhances the remobilization of non-quantified masses of bottom sediments, thus inhibitinginorganic toxicants from reactive processes. Furthermore, the periodical dredging of the navigation channels causes intense remobilization of sediments. Meanwhile, a progressive green coverage of several areas of the mudflat could be witnessed. The sea grass *Zostera noltii* was then identified in the course of an exploratory sampling initiative and appeared to be the residence substrate for small gastropods. There is also evidence about the presence of squids.

In sum, there are several factors potentiating the presence of radionuclides and heavy metals in the estuarine bay, which, in turn, contaminate the food-chain that ultimately impacts human health. The robotic team herein presented is expected to foster accurate spatio-temporal sediment and biota sampling so that environment researchers can study these phenomena in detail.

### 3.2. Mission Workflow

**Figure 3:**
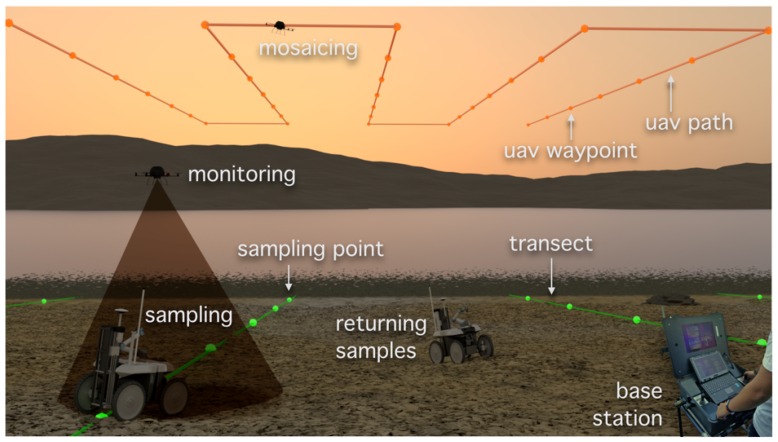
Diagram representing the sediment sampling mission workflow.

This section presents a mission workflow (storyline) devised for the case study, taking into account the characteristics of the presented robotic system. Figure 3 illustrates the mission workflow, which assumes the presence of a human expert in a base station by the operations’ site. This human expert plays three roles throughout the workflow. Firstly, the expert must plan and supervise the mission through a Graphical User Interface (GUI). Secondly, the expert must be available to recharge the robot with empty sample containers whenever required. Thirdly, the expert must accommodate the samples and take them to the lab for off-line processing. Hence, the expert works in parallel with the robot to fulfil the mission.

The mission workflow is defined according to the following enumerated steps:

1. The human-robot team reaches the operations site. The robots are unloaded from the transportation vehicle. Based on satellite imagery of the operations’ site, the human expert defines the workspace boundaries. These boundaries will be used by the robotic system to constrain its operation range.
2. The aerial robot takes off and performs a scan to cover the workspace defined by the expert. As a result of these scanning procedure, the UAV builds a high resolution geo-referenced mosaic from a set of mutually-registered aerial images. The mosaic is then presented to the expert, which is now able to discard the satellite imagery, as it is most likely outdated.
3. At the base station, the expert segments potential key features of the environment, such as water ponds and channels, sea grass coverage, salt marsh vertical vegetation, sand banks and all sorts of physical obstacles to the UGV’s navigation. Then, based on this meta-data, the expert specifies a set of transects to be sampled by the robot (see Section 3.3).
4. With the information collected in the previous step, the ground vehicle is tele-operated by the expert so as to traverse the transects and periodically sample the terrain while avoiding any peril in its way. At each sampling point, the expert requests the ground vehicle to perform a sediment sampling behavior. If intended by the expert, the aerial vehicle is used to provide aerial images, augmenting the operator’s perception about the mission execution.
5. When the ground vehicle has either its sample containers filled or has visited all of the defined sampling locations, the expert tele-operates the robot back to the base station.
6. Back at the base station, the expert unloads the sample containers into isothermal boxes with cooling pads, which are subsequently brought to the lab for post-processing. If the mission is not complete, the expert loads the ground vehicle with empty sample containers and resumes the mission (return to the previous step).
7. Once the mission in the current operation’s site is complete, the expert washes the ground vehicle with fresh water so as to remove dirt and salt residues. Then, the expert may be called upon to execute some maintenance procedures, such as recharging batteries or re-inflating tires. Finally, the human-robot team leaves the operations site.

### 3.3. Sampling Procedure

Mission control’s Step 3 requires the expert to define the sampling procedure. This is a critical point in the process from an environmental monitoring perspective. Failing to produce the proper sampling procedure results in a failed mission.

A proper sampling campaign must ensure that samples are representative of the spatial diversity of the mudflat. Furthermore, the sampling campaign must also grant that the sampled volume is sufficient for the subsequent off-line analysis to provide significant results. In routine monitoring situations, where the density of contaminants per sample is expected to be low, a possible approach to overcome detection difficulties (e.g., detection limits in gamma spectrometry for natural radionuclides) is to collect large sample volumes for further concentration of the target analytes. As a consequence, large sample volumes are required if contaminants are to be detected therein. This is an important issue that must be taken into account when selecting the scale of the robotic system.

The sampling procedure follows the principle of transect sampling generally described by the International Commission on Radiation Units and Measurements, for the purpose of estimating spatial distribution patterns of radionuclides in large areas with closely-spaced sampling locations [3]. Sediment cores should be extracted both in bare and sea grass-covered mudflat, to support a posteriori partition analysis of metal and radionuclides in sediments and sea grass. For a large-scale real-life sampling campaign, 500 m-long transversal transects should be defined perpendicularly to the shore line. Along these transects, the ground vehicle should stop every 100 m along the transect. Each stopping position defines the center of a circular sampling area with 6 m in diameter, from which nine sediment cores should be randomly extracted by the robot. Due to practical reasons, a smaller scale protocol was followed in the field trials described in Section 6. Real-life campaigns to be engaged in the future are expected to run the full protocol.

One major issue in sampling the bottom sediments of estuarine environments is that the time frame available for sampling is constrained by tidal activity. In general, the sampling procedure must be within a time span of approximately 4 h. Furthermore, the Tagus’s estuary in the Alcochete region, where the field trials took place, is mostly flat, and so, the water level covers/uncovers the mudflat at a very fast pace. Due to all of these reasons, the initial sampling points need to be the ones that are farther from the shore. Then, progressively, sampling can proceed in the direction of the shoreline.

### 3.4. Sampling Processing

At the lab, the cores are unfrozen and sectioned in depth layers (0 cm–5 cm; 5 cm–15 cm; 15 cm–25 cm; 25 cm–35 cm; 35 cm–45 cm), and all sections of a specified depth range are mixed into a composite sample. Composite samples from specified depth layers are oven dried at a temperature of 60°C. The fine grain size fraction composed of silts and clays is separated from sand particles in a mechanical sieving system (silt, clay <64μm, sand <200μm). Samples are kept dried in tagged plastic containers for further radiological and trace metal analysis.

## 4. The Robotic Team

This section details the mechanical, hardware and software designs of the two vehicles comprising the robotic team, devised to meet the requirements of the proposed experimental protocol for robotic sampling of estuarine mudflats.

### 4.1. The Unmanned Ground Vehicle

#### 4.1.1. Mechanical Hardware

The UGV’s components have been carefully selected to meet the requirements of advanced mobility in estuarine mudflats. This type of environment poses serious difficulties in terms of robot mobility. The robot needs to be able to overcome water streams, move in dry and wet sand and several levels of muddiness and tackle vegetation, rocks and other debris. Moreover, to be able to place the drilling tool in the desired sampling point, it is necessary to have a fine control over the robot’s pose, meaning that the robot needs to be highly maneuverable. In addition to the mobility aspects, the design of the mechanical platform took into account that salt water will wear out materials, that the presence of mud and sand may jam mechanical joints and that the robot will be subject to wide temperature ranges.

**Figure 4:**
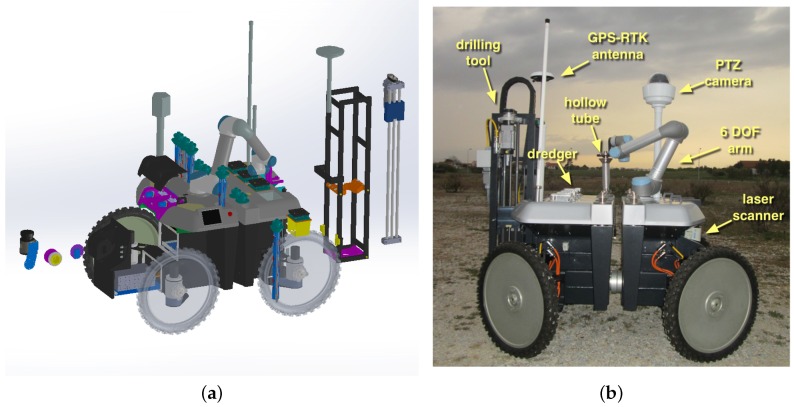
The Unmanned Ground Vehicle (UGV). (**a**) An exploded CAD view of the UGV; (**b**) the UGV turning around its geometric center.

Figure 4a depicts the UGV and its CAD exploded view. The UGV is 1100 mm × 1525 mm × 1326 mm (width × length × height) and weighs 240 kg when fully loaded. It is composed of two main components, (1) the mobility platform and the (2) drilling tools, which can be replaced depending on the tasks at hand. The platform is a rugged version of the all-terrain robot INTROBOT [2].

The robot’s body is made of aluminum and composite materials. The aluminum was the chosen material for the chassis due to its light weight, strength, corrosion resistance, flexibility, thermal conductivity, ductility and cost efficiency. Furthermore, it enables the construction of a fully-sealed fan-less system without overheating issues. To further extend life expectancy and resistance to extreme environments, the robot was coated with a hydrophobic paint. This special cover also repels dust and mud, reducing corrosion, common in maritime environments.

The robot is split into a frontal block and a rear block, each actuated by two wheels. These two blocks are attached via a passive longitudinal joint. The passive joint improves the four wheels’ contact with the ground, even on uneven terrain, or when the robot needs to overcome some small obstacles (see Figure 1a). Locomotion is accomplished by four-wheeled independent transmission blocks in a no-slip quasi-omnidirectional locomotion structure known for its low mechanical stress and odometry errors [41]. Each transmission block contains a 250-W Merkes motor and a 550-W Parker motor for steering and traction, respectively.

With its independent steering and traction motors, the robot is able to move in Ackerman and double Ackerman configurations, as well as to rotate around its own geometric center. This multi-modal locomotion becomes of special importance whenever the robot needs to make fine adjustments to its pose to, for instance, align its drilling tool to a specific sampling spot.

The wheels are 60 cm in diameter and are a combination of aluminum rims with motocross tires of 10 cm in width. With this configuration, the robot exhibits a considerable ground clearance, which is key to handle small obstacles in the terrain. All tires are partially deflated to half of the recommended pressure to increase the footprint and reduce the chances of slippage. Each motor is directly coupled to its corresponding wheel through a gear. The absence of chains or belts not only reduces energy loss, but also the number of failure points. An issue often neglected in current robotic platforms is the ease of their transportation and storage. To facilitate transport, each wheel supports hand locking and manual clutch. The longitudinal passive axis can also be locked in the central position.

**Figure 5:**
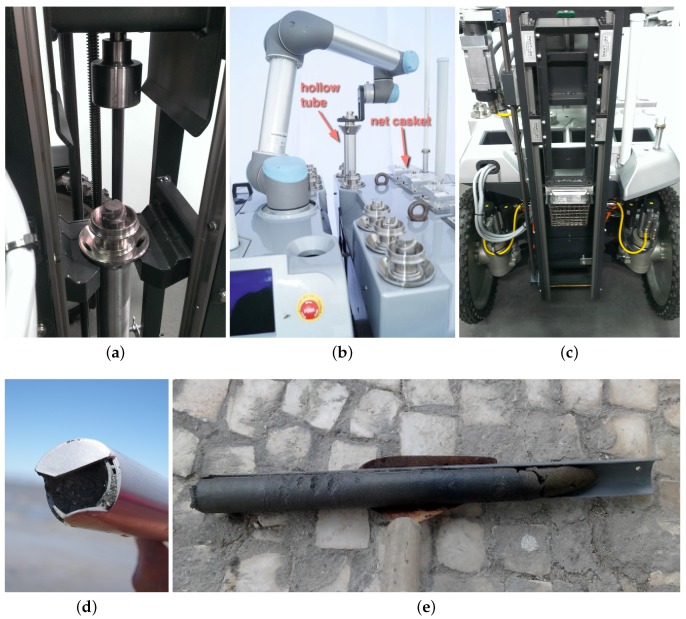
The drilling and dredging tools. (**a**) A hollow tube being attached to the drilling tool; (**b**) the robotic arm grasping a hollow tube to insert it into the drilling tool; (**c**) a dredger being pushed downwards by the dredging tool; (**d**) the hollow tube’s tip designed to prevent the core from slipping during extraction; (**e**) a sediment sample laid on the PVC half-tube after extraction.

On the top of the mobility platform, the UGV is equipped with a rectangular tube frame, underneath the composite covers. This frame provides the structural strength necessary to hold nine sample containers, a robotic arm to manipulate them and the drilling tool. An actuator in the drilling tool revolves a cylindrical hollow metallic tube with an internal section of 45 mm and a length of 500 mm, which is simultaneously pushed downwards by a linear actuator. Figure 5a shows a hollow metallic tube placed in the drilling tool ready to initiate a sampling procedure. At the end of the drilling process, the hollow tube will contain a cylindrical sediment sample. To avoid the contents of the hollow tube falling into the ground, the tip of the tube has a wedge (see Figure 5d). The whole drilling process takes approximately 60 s, which is roughly the same time a human expert spends handling a manual tool.

To cope with the contingency of drilling in surfaces with buried hard elements (e.g., rocks), the linear actuator’s current and position is monitored continuously throughout the process. Once the available containers are filled, the sediment samples in the hollow metallic tubes must be removed as a pack, that is without mixing sediments from different depths. To facilitate this task, each hollow metallic tube has two half-hollow PVC tubes (separated longitudinally), which easily slide out of the metallic tube with the help of the human expert. Figure 5e shows a sediment sample collected with the presented system. As is possible to see, the sample is compact and fills the PVC half-tube almost entirely.

At each sampling point, it is necessary to store the filled hollow tube in its storage socket and place an empty one in the drilling tool. This process is carried out autonomously with an Universal Robotics UR5 compliant robotic arm. With six-DOF, this robotic arm is able to move the tubes between storage socket and drilling tool without collisions. Moreover, the arm has compliant properties, which is important to ensure the safety of people working in the robot’s workspace. Figure 5b depicts the robotic arm while picking an empty hollow tube from its storage socket.

The drilling tool works in a plug-n-play fashion in order to facilitate accommodation and transport. This also allows interchanging sampling tools. For instance, the robot can also be equipped with a tool for dredging seaweed. In this case, the tool also uses a linear motion to push a 1.4 cm${}^{3}$ grid-like dredger towards the soil, which is then filled with seaweed and clams by moving the robot forward. Figure 5c depicts the robot equipped with the dredging tool pushing a dredger towards the soil. In this paper, we do not exploit this tool in the experimental protocol.

#### 4.1.2. Electronics Hardware

Field robotics is often limited by the energetic autonomy that robots exhibit. Energy harvesting is an interesting solution to ensure long-lasting operation. However, in our case, given that the robot’s operation depends on the tides, there is little opportunity for battery recharging cycles. As a consequence, the robot must be equipped with sufficient energy for a complete mission. In this sense, energy is supplied by eight lithium ion cells with a total capacity of 100 Ah. Lithium is known by its energy:weight ratio, which combined with the EMUS intelligent Battery Management System (BMS), offers more than 4 h of operation. The BMS extends cell life by keeping voltage, current and temperature parameters within manufacturer ranges.

**Figure 6:**
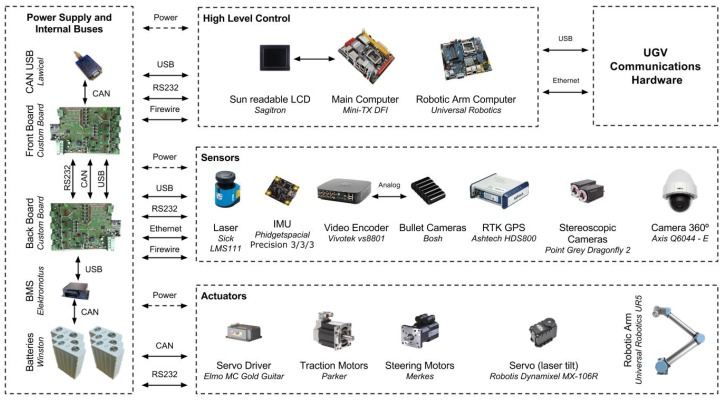
UGV’s modular hardware architecture. Dashed arrows represent power supply connections. Solid arrows represent device-device intra-robot wired communication links (e.g., RS232, Firewire, CAN, USB, Ethernet). The UGV communications hardware block abstracts the components for wireless communications, whose details are discussed in Section 5.2.

The usability of any mechanical platform relies on the selection of a proper set of components. They are responsible for efficient energy management, for low level motor control, for providing sensory feedback to the control system and for ensuring reliable communications with the base station. Figure 6 depicts the modular hardware architecture that equips the UGV, made from off-the-shelf components offering a close to plug-n-play advantage.

The main processing unit combines an Intel Core i7 CPU with a mini-ITX industrial board, ready for extended temperature ranges. Connected to it, two custom-built printed circuit boards, each one with its own micro-controller, are responsible for distributing and controlling supply voltages across all electronic components. These boards also provide internal buses for RS232, CAN and USB intra-robot wired communications. On-board current monitoring of each power output allows for continuous information on devices’ consumption and precise power management, while aiding on early problem detection. A total of 15 analogue and digital I/O ports is available through the two boards for health monitoring sensors (e.g., temperature, vibration). The Universal Robotics UR5 compliant robotic arm includes its own processing unit, which is connected to the UGV’s main computer through an Ethernet connection.

The hardware responsible for maintaining wireless communications between UGV, UAV, base station and human-robot interaction devices is detailed in Section 5.2.

For precise pose estimation, the UGV relies on a Phidgets Inertial Measuring Unit (IMU) and on an Ashtech HDS800 high precision GPS-RTK, which provides centimeter-level accuracy based on GPS corrections obtained from the RENEPnetwork via GPRS. To provide the user with good situational awareness, the robot is equipped with a set of body-fixed Bosch Color Mini Bullet cameras. Three of these cameras are aligned with the forward robot motion; a fourth camera is pointing backwards; and a fifth camera is looking downwards to the drilling tool. To provide higher flexibility to the user, the robot is also equipped with an Axis Communications Q6044-E PTZ camera. The video output of these cameras feeds a Vivotek VS8801 video encoder, which sends video streams using the Real Time Streaming Protocol (RTSP) to the base station through the wireless network. Although autonomous navigation is not addressed in this paper, it is worth mentioning that the robot is equipped with distal sensors for environment perception. A SICK LMS111 2-D laser scanner is mounted on a Robotis tilting unit for delivering complete 3D point clouds. To complement the laser scanner, a pair of PointGrey Dragonfly2 cameras is available for depth estimation. Autonomous behavior based on 3D sensory data has been studied in the UGV’s predecessor, INTROBOT [42].

Both sampling tools (i.e., for drilling and dredging) are built based on the same base equipment, simplifying both software and hardware implementations. Namely, 550-W Parker motors are used and controlled by Elmo Gold Guitar controllers. Limit switches in the linear actuators are used to avoid overheating the system in case any control problem arises. Hengstler encoders and a laser pointer are used to report the exact position of the tool with respect to the ground. This allows the system to adjust the drilling height in the face of uneven terrain. Internal power consumption and temperature sensors in the motor controllers inform the control system regarding the mechanical stress being induced in the sampling tool. For instance, a power consumption peak informs the control system about the presence of an obstruction to the drilling process, such as a small buried rock.

#### 4.1.3. Control Software

To promote scalability, adaptability and interoperability, as new requirements are presented to the robotic system, the software layer responsible for controlling the UGV runs on top of the Robot Operating System (ROS) [43]. ROS provides a publish-subscribe inter-process messaging service on top of a master-slave communications framework. To avoid a single point of failure, a multi-master configuration is used by including the rosbridge extension [44]. Additionally, rosbridge provides a JSON API to ROS functionality, which enables interoperability between robots and the base station over web-based communication channels. Another strong point in favor of ROS is its wide acceptance by the robotics community, meaning that new algorithms and device drivers are frequently made publicly available and, then, easily integrated into ROS-based robots.

At each moment, the system must gather sensory feedback about the robot’s pose and its surroundings. This sensory feedback comes asynchronously and, therefore, must be associated with a time stamp. ROS provides this functionality. Based on the sensory feedback provided by the wheel odometers, by the GPS-RTK device and by the inertial measurement unit, an Extended Kalman Filter (EKF) recursively estimates the UGV’s optimal pose. Due to the absence of tall buildings or trees in estuarine environments, the GPS-RTK device is capable of producing centimeter-level global localization accuracy alone, in most situations. Nevertheless, wheel odometry helps the EKF to filter out GPS-RTK spurious noise and signal drop-outs. To mitigate motion estimate errors resulting from wheel slippage or blockage, we estimate the motion of the robot using each wheel independently and then fuse the four estimates in a weighted manner. The more similar the motion estimated from one of the wheels is from the estimates obtained from the other three wheels, the higher is its weight in the fusion process. This process exploits the redundancy introduced by the four wheels to disregard deviating motion estimates. Please refer to [41] for further details.

**Figure 7:**
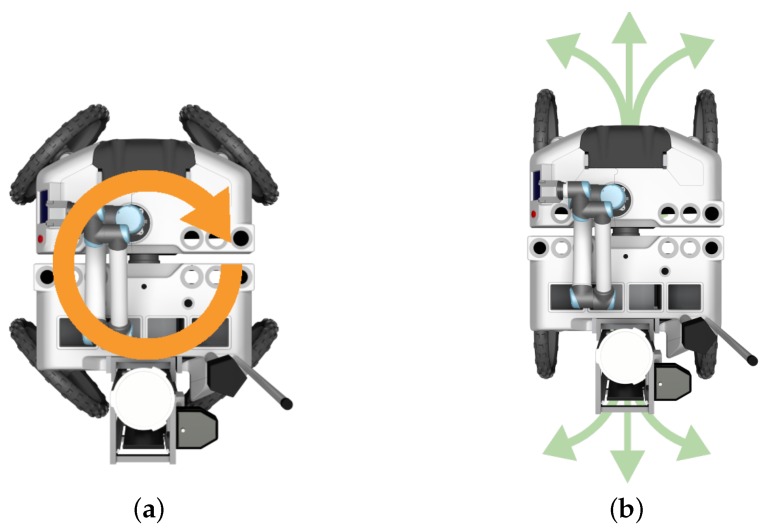
UGV’s possible motions attainable by the robot in two different locomotion modes. (**a**) The orange arrow depicts the type of motion that the robot can attain when in the turning point; (**b**) the green arrows depict the type of motion that the robot can perform with double Ackerman locomotion.

Controlling the motion of a four-wheeled steering robot in uneven terrain is a complex task, as, ideally, the four wheels should be synchronized and should behave in a compliant way with respect to the environment in the face of transient external forces [41,45]. As in mudflats, wheels get partially buried often and, hence, are subject to non-transient external forces, a compliant behavior would hamper the robot from promptly reacting to the expert’s requests. Therefore, to control the four wheels, the UGV simply tracks, with decoupled stiff PID controllers, the traction speed and steering angles kinetically defined by the desired locomotion mode (e.g., double Ackerman, turning point). The control system also selects the locomotion mode that is best suited for the given motion command. A motion command, which can be obtained from the user via one of the tele-operation interfaces, is defined in terms of a tuple of linear and angular velocities. If no linear velocity is requested, then the control system switches to the turning point locomotion mode (see Figure 7a). In this mode, the robot will turn without performing any displacement. If both linear and angular velocities are requested, then the control system switches to double Ackerman locomotion mode (see Figure 7b). However, if the arc of trajectory resulting from the desired linear and angular velocities is infeasible, i.e., below a radius of 0.85 m, then the robot opts for the turning point locomotion mode.

The control system also encompasses a node responsible for controlling the drilling tool and another for controlling the robotic arm. While the drilling tool needs to be controlled at the actuator level, the robotic arm is abstracted via the MoveIt! API [46]. With MoveIt!, the robotic arm trajectory is guaranteed to be collision-free, given a 3D model of the UGV and the target pick-and-place points. Given that the robotic arm’s workspace does not change during the mission, trajectories are pre-planned and then play-backed whenever required. This approach allows saving computation and speeding-up the sampling procedure. The nodes responsible for controlling the drilling tool and robotic arm are available as services in the ROS network for asynchronous invocation through the graphical user interface. This way, the expert is able to request the system for a drilling sequence and fully control which container is used at each sampling point.

### 4.2. The Unmanned Aerial Vehicle

#### 4.2.1. Mechanical, Hardware and Control System

The aerial team member, the UAV, was designed to withstand the difficult operational environment of estuarine environments, where robustness and reliability are key. Taking this into account, a six-rotor configuration with vertical take-off and landing capabilities was chosen (see Figure 1). This configuration combines a good thrust-to-weight ratio and redundancy to enable emergency landings in the event of a malfunctioning motor. The UAV’s frame is made of a mix of aluminum, copper and composite materials weighing 4.30 kg when fully equipped.

**Figure 8:**
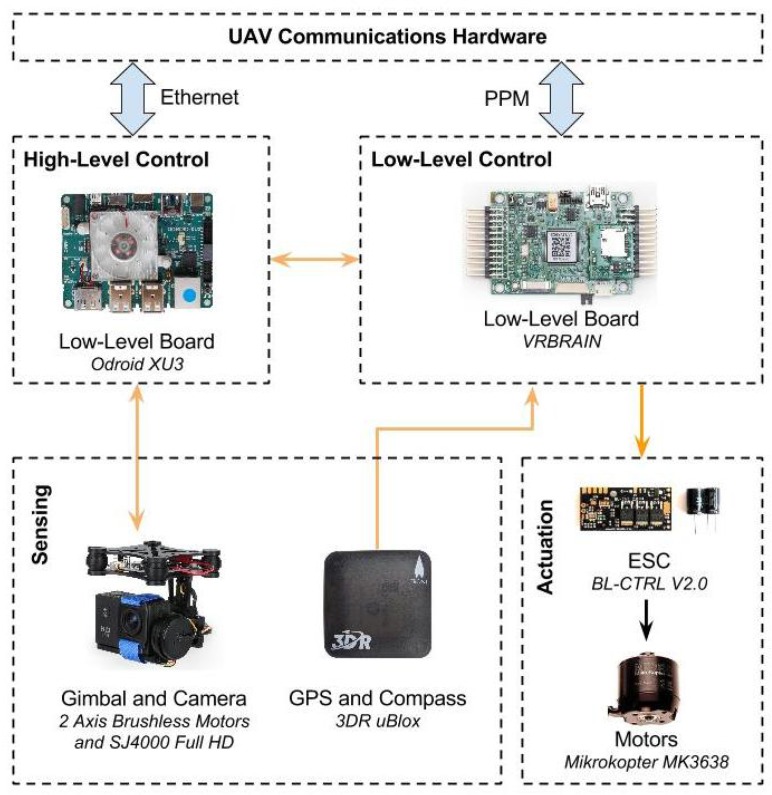
The UAV’s hardware architecture. Orange arrows represent serial connections between low-level and high-level control boards, sensors and actuators. The UAV communications hardware block abstracts the components for wireless communications, whose details are discussed in Section 5.2. This block communicates with both high-level and low-level boards through Ethernet and PPMconnections, respectively, represented with blue arrows.

An overview of the UAV’s hardware architecture, components and connection types is depicted in Figure 8. Propulsion is ensured by six 35-A BL-CTRL V2.0 Mikrokopter brushless motor speed controllers, which power six Mikrokopter MK3638 brushless motors. Each motor has a carbon fiber propeller measuring 13 inches (≈33 cm) in diameter, with a 6.5 inch (≈16.5 cm) pitch. This combination of motor, propeller and ESCis capable of 2 kg of thrust at 26 A, to a total of 12 kg, which gives the UAV a 2.7:1 thrust-to-weight ratio. The UAV is power supplied by a Maxamps LiPo 12000XL 4S 14.8 V, which allows flight times of around twenty minutes.

**Figure 9:**
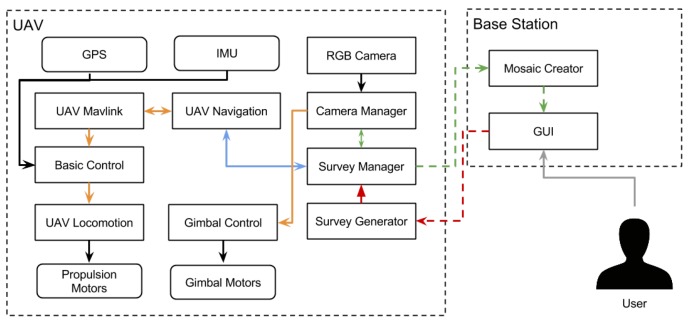
The UAV’s software architecture. The rounded rectangles depict low-level actuators and sensor, while the others depict the software modules. The red arrows depict messages with geo-referenced information, with the dashed line depicting the points that form the aerial survey area and the filled line the next point for the UAV. In green are depicted the images exchanged between the software modules; the filled line is the camera’s raw data and the dashed line the images used for the aerial mosaic. In orange are depicted the action messages and in black the sensor and actuator streams. The blue arrow depicts the action’s sent to the UAV’s navigation in order to perform the aerial survey.

The UAV’s control system is supported by two computational units, one strictly dedicated to low-level motion control and the other to high-level mission execution functions. Wired communication between controllers is assured by the MAVLink Micro Air Vehicle Communication Protocol via a serial link. The low-level control unit is a VRBrain from Virtual Robotix, interfaced with an on-board IMU based on the MPU6500, a MS5611 barometer and an external GPS device from Ublox. The basic navigation and low-level stabilization is assured by a modified version of the open-source Arducopter platform [47], whereas high-level navigation and interaction features are handled by dedicated ROS nodes (see Figure 9). Sensor fusion is performed by and Extended Kalman Filter (EKF) that estimates the UAV’s pose with measurements of velocity and angular orientation based on the rate of gyroscopes, accelerometer, compass, GPS, airspeed and barometric pressure. In the current implementation, the UAV fuses inertial measurements with 2D velocity and position from GPS and barometric pressure. The high-level functions are ROS-enabled and run on top of Xubuntu’s 14.04 lightweight Linux distribution. The computational unit supporting high-level functions is an Odroid-XU3 from Hardkernel equipped with an Exynos octa-core CPU. To take aerial images, which support the creation of aerial mosaics for a proper mission planning, the UAV uses a SJ4000 camera with diagonal 170° field-of-view mounted on an active gimbal.

The hardware responsible for maintaining wireless communications between UAV, UGV, base station and human-robot interaction devices is detailed in Section 5.2.

#### 4.2.2. Flight Behavior and Mosaic Creation

**Figure 10:**
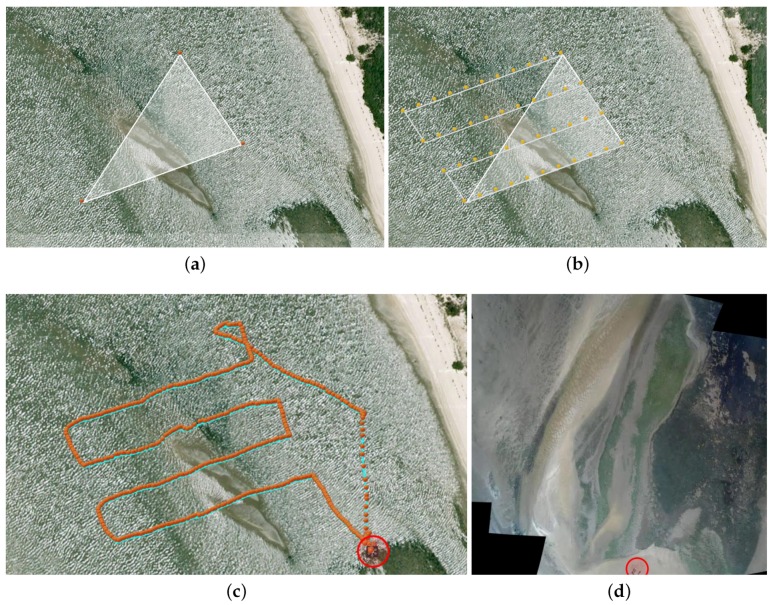
The aerial mosaic creation process. (**a**) The expert defines the survey area by setting three geo-referenced points in the GUI (depicted in orange); (**b**) a set of waypoints (in yellow) is automatically generated so as to ensure that the UAV takes significant key frames for the registration process; (**c**) satellite imagery of the site with the UAV’s executed path overlaid. This path was generated by following several waypoints in sequence. The UAV’s starting spot is depicted by the red circle overlay. (**d**) The resulting aerial panorama with the UAV’s starting point depicted by the red circle overlay.

As satellite imagery is mostly outdated, it cannot be used by the expert to precisely define the sampling mission. For this reason, the UAV, in cooperation with the human operator, is used to produce an up-to-date aerial mosaic of the operations’ site. To perform the aerial survey, the expert needs first to specify the UAV’s workspace by means of three geo-referenced markers on top of satellite imagery (see Figure 10a). These markers define a parallelogram where the UAV will perform a line-sweep pattern with parallel lines running across the area to be mapped (see Figure 10b).

The mosaic creation behavior is then responsible for driving the UAV through a set of waypoints so as to cover the area defined by the expert. These waypoints are automatically defined according to the camera’s Field Of View (FOV), learned from calibration and the required level of inter-image overlap required for a proper image registration process. Concretely, the system uses the FOV of the UAV’s camera to estimate the area covered by each image at different altitudes. Then, given the survey area and image overlap selected by the operator, the system calculates the waypoints required for the aerial mosaic creation. Although this process is automated, the operator can always fine tune the system for a specific application by changing the desired altitude, image overlap and UAV’s velocity. While, ideally, very large areas could be mapped with high detail by flying at low altitude, the resulting large flying missions are hampered by the UAV’s limited energetic autonomy. To prevent the operator from creating infeasible surveys, the system overrides the altitude, speed and image overlap parameters to be able to cover the selected area. For the current hardware configuration, the UAV typically travels at an altitude of 50 m. At each waypoint, an image is recorded and stored in the high-level processing board. Once the flight is complete, all images are transferred in batch modeto the base station so that the mosaic can be built and displayed to the expert. With the updated imagery, the user can define with greater accuracy the sampling points for the UGV. The mosaic is built using the open-source software Hugin Panorama [48].

Figure 10 depicts the several phases involved in the mosaic creation process, during one of the field trials. The differences between the satellite imagery and created mosaic are quite remarkable. It is even difficult to visually map both. This is caused by the significant changes that estuarine environments suffer across seasons and, even, on a daily basis. Without the aerial mosaic, the expert would not be provided with a sufficiently up-to-date aerial perspective of the environment and, thus, would not be able to properly plan the sampling mission.

## 5. Human-Robot Teamwork

As defined in the mission workflow of the experimental protocol (see Section 3), the robots and human expert need to interact for the fulfillment of the mission. These interactions require a communications infrastructure, a set of I/O devices, Graphical User Interfaces (GUI), a diagnostic system capable of informing the human expert of unexpected issues and some coordination logic for the implementation of interaction patterns between human and robots. The following sections describe these elements.

### 5.1. Human-Robot Interaction Devices

**Figure 11:**
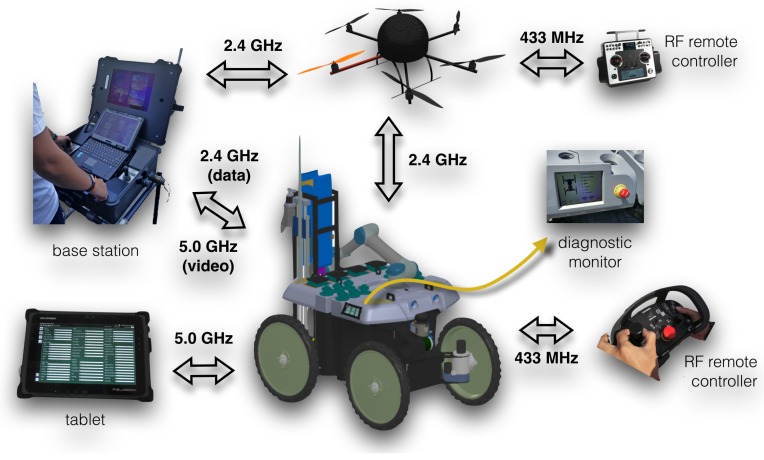
Human-robot interaction devices and teamwork underlying communication channels.

The human expert is allowed to interact with the robotic system through (1) short-range radio frequency (RF) controllers; (2) a wireless tablet; (3) a wireless portable base station; and (4) a diagnostic monitor attached to the robot (see Figure 11). The following details each interaction device and underlying communication infrastructure.

There are two dedicated RF controllers, one for the UGV (a custom Datek device) and another for the UAV (a Graupner MC-32 device), both useful to ensure fine motion control in the line of sight. In the specific case of the UGV, the RF controller can also be useful to drive the robot towards a specific spot that the expert has identified while inspecting the environment by foot. In the UAV case, the RF controller is mostly useful for tuning flight controller parameters and providing basic functionality in case of emergency.

Through the tablet, a FieldBook I1 equipped with a touch screen, the user is able to move closer to the operations site while still having almost all of the functionality available at the base station. This flexibility is important as real-life environmental monitoring campaigns often require the expert to freely move in the environment.

The portable base station allows the operator to gain access to all graphical user interfaces available to the system, allowing the user to configure and execute the environmental monitoring mission. The base station is composed of a rugged laptop, two analogue joysticks from CTI, a sunlight readable display from Sagitron, a digital video decoder and a wireless router attached to a high-gain antenna (see below). The rugged laptop is a Getac V200 with an Intel Core i7-620LM 2.0 GHz processor and 4 GB of RAM. The joysticks enable the manual control of the UGV’s locomotion (linear and angular velocities) and its pan-tilt-zoom camera individually. The 1200 GB cd/m${}^{2}$ sunlight readable display shows the video streaming of the UGV’s on-board cameras for the operator to have a clear notion of the surroundings of the robot. The mentioned video streams are encoded in the robot and transmitted to the base station using the Axis VAPIX API [49]. This API allows one to choose between streaming over HTTP or RTSP. The first works by transmitting individual JPEG video frames at a fixed rate or by request, and it was initially preferred to ease the acquisition of images for post-processing (e.g., to identify obstacles/vegetation/water or to overlay telemetry data). Results, however, show that this solution would consume a large amount of bandwidth, at the point of being unusable in wireless communications. Thus, the system currently encodes and decodes video streams using the RTSP protocol, which is more suitable for real-time video transmission over the network at the cost of: introducing additional lag, due to buffering; and restricting the ability to process individual frames, due to video compression.

### 5.2. Communications Infrastructure

The distributed nature of the human-robot teamwork demands for heterogeneous wireless networks. This section describes the several elements that compose the communications infrastructure of the proposed system (see Figure 11), that is, the UGV communications hardware and the UAV communications hardware blocks depicted in Figure 6 and Figure 8, respectively.

**Figure 12:**
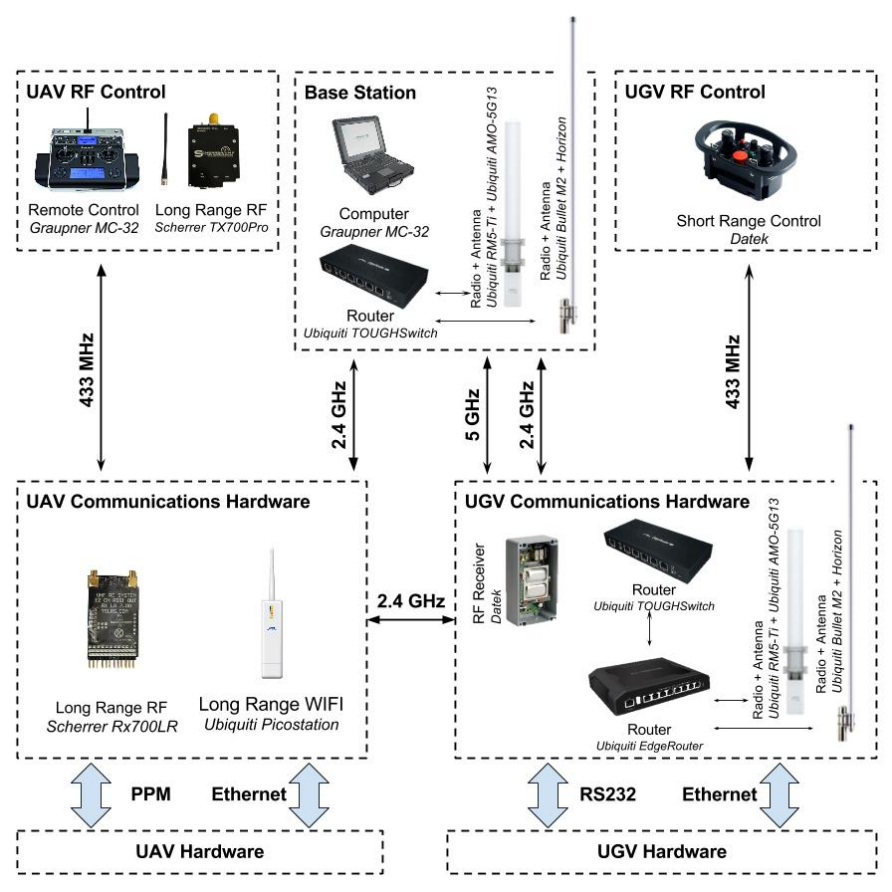
Communications hardware architecture for both the UAV and UGV. Black arrows represent the wireless connections between interaction devices (e.g., remote RF controllers), the base station, UAV and UGV. Blue arrows represent the wired connections between the UAV’s and UGV’s communications and control hardware (see Figure 6 and Figure 8).

The UGV accesses the wireless LAN through two high gain (13 dBi) 2.4-GHz Horizon IH-G12-F2324-V antennas and two high gain (12 dBi) 5.0-GHz Ubiquiti AMO-5G13 antennas (Figure 12). This dual-band antenna configuration ensures redundancy, allowing multiple connections with minimal interference. To reduce communications latency, which is key for stable robot tele-operation, video streaming from the UGV’s on-board cameras is done through the 5.0-GHz link, whereas low-volume telemetry and coordination data are transmitted over the 2.4-GHz link. The UGV internal Ethernet comprises a Ubiquiti Gigabit Edge Router and a Ubiquiti Tough Switch connected to two radio transmitters, a Ubiquiti Rocket M5 and a Ubiquiti Bullet M2, which are, in turn, connected to the four high gain antennas. With this configuration, the UGV is able to communicate with other devices within a line of sight radius of 1 km with transmission rates of up to 150 Mbps. The line of sight assumption holds in mudflats due to the absence of large obstacles (e.g., buildings, trees, slope variations). A GSM up-link is also available as a backup to wireless drop-outs.

The UAV accesses the wireless LAN with a 2.4-GHz Ubiquiti Picostation 2HPa, which is a radio transmitter with integrated antenna. With this configuration, the UAV is able to communicate with other devices within a line of sight radius of 500 m with transmission rates of up to 54 Mbps. The UAV’s communications range can be expanded by connecting directly to the UGV, serving the latter as a communications relay.

The human-robot interaction devices communicate with both the UGV and UAV with the following wireless equipment. The base station is equipped with a Gigabit Edge Router and wireless LAN communications hardware identical to the one employed in the UGV. The UGV’s RF controller communicates with the robot through a 433-MHz custom Datek device with range, in line of sight, of up to 250 m. Communications between the UAV’s RF controller communicates and the robot are maintained through a 433-MHz Scherrer Rx700LR+PSU receiver and a 433-MHz Scherrer TX700Pro transmitter with three power stages, 500 mW, 1 W and 2 W, allowing theoretical ranges of up to 100 km.

#### System’s Health Supervision

One of the most important capabilities in complex robotic systems is the ability for the system to pro-actively inform the human operator regarding the system’s overall health. In fact, when the number of components and subsystems increases, the more important it is to identify potential points of failure in the early stages of occurrence during mission execution. A health monitoring supervision system requires two modules: (1) a versatile diagnostic system; and (2) an associated graphical interface. The following describes these two modules.

In the robotic system herein presented, a custom implementation of the ROS Diagnostic Stack, which comprises tools to collect, analyze and view diagnostic information (e.g., the state of the motors, communications throughput or energy consumption), assures that all relevant data are collected and tagged for efficient processing. The system software packages, in particular the ones that communicate with hardware components (e.g., device drivers, communications), are attached to diagnostic routines using the ROS Diagnostic Updater API. These diagnostic routines execute independently at predefined frequencies and are configured individually, enabling access to a set of indicators that are specific for each component or subsystem. Collected diagnostic information is then sent to the ROS diagnostic aggregator, which is responsible for grouping and categorizing data according to pre-set rules.

To exemplify, let us analyze the kind of diagnostic information that is produced by three key nodes. The motor control node reports to the diagnostic aggregator, at 1 Hz, the motor controllers’ temperature and current consumption. The Battery Management System (BMS) reports to the diagnostic aggregator, at 0.5 Hz, the overall battery level, the instantaneous power consumption and the voltage per cell, among others. The communications monitor node reports to the diagnostic aggregator, also at 0.5 Hz, the wireless signal strength and noise level, the transmission and receiving rates, among others.

Error states are also immediately reported when monitored variables reach some pre-defined thresholds (e.g., the total battery charge being below 5% of the total charge). Similarly, there is diagnostic information associated with tele-operation control, communications, pose estimation, robotic arm control, on-board computer, among others. In fact, by relying on the ROS diagnostic stack, new diagnostic routines can be added when needed, without interfering with the already existing ones, making the system scalable and robust when evaluating the health of the system.

**Figure 13:**
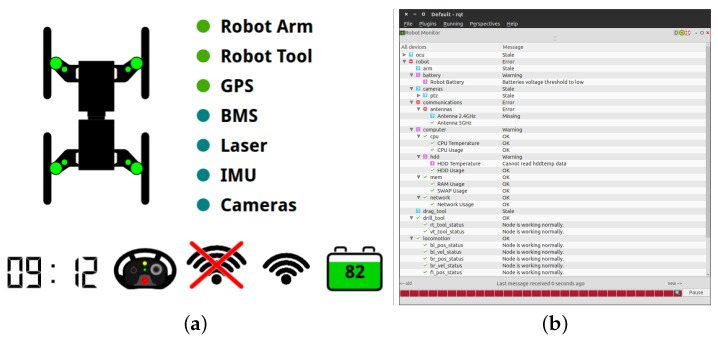
The diagnostic tool. (**a**) Graphical summary of the system’s health; (**b**) detailed diagnostic information. In this case, the system is reporting an issue related to one of the antennas.

A graphical summary of the diagnostic information is provided to the operator at runtime through a GUI available at the tablet and base station (see Figure 13b). Detailed information about the overall system’s health and potential points of failure is also available to the expert through the diagnostic monitor attached to the robot (see Figure 11 and Figure 13a). The diagnostic information can also be consulted at a later time using the ROS Diagnostic Analysis tool, which converts all of the information collected into .csv files.

### 5.3. Mission Graphical User Interface

**Figure 14:**
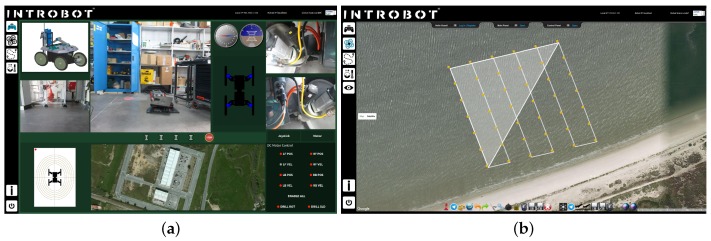
Mission control graphical user interfaces. (**a**) UGV’s tele-operation view; (**b**) UAV’s aerial survey configuration view. In orange are depicted the geo-referenced points that define the area of interest while in yellow the waypoints the UAV needs to follow during the survey.

The whole system is built upon ROS, meaning that nodes can be easily deployed and interfaced in a seamless way. This is particularly useful to enable the implementation of graphical user interfaces that can be visualized across the network. Figure 14 depicts the graphical interface of the mission control tool, which was written in C++ using the Qt framework. The tool provides two main views. The first view allows the operator to tele-operate the UGV using the two joysticks and, simultaneously, visualize telemetry data (e.g., robot’s pose) alongside the video streams produced by the UGV’s on-board cameras.

The second view allows the operator to define the aerial survey as describe in Section 4.2.2. The main component of this view is the web-based application linked with the Google Maps API. This API was selected for its built-in geo-referenced and reconfigurable visualization features, essential for a user-friendly robot behavior specification. The developed interface includes a system for users authentication, a system for path XML file management, live tracking of the current position of each robot overlaid on satellite imagery and live visualization of key telemetry data (e.g., snapshots taken by the on-board cameras). The application was constructed as a Java Server Page assembling JavaScript for the interface with the Google Maps API, JQuery Library for the addition of control panels and AJAX for the implementation of user-management features. The interface with the multiple robots in the ROS network is ensured by the JSON API provided by rosbridge.

### 5.4. Human-Robot Interaction Patterns

According to the mission workflow defined in the experimental protocol proposed in Section 3, the system’s operation is split into two main phases: (1) mission preparation; and (2) mission execution. Each phase imposes different interaction patterns on the human-robot team. The following describes each of these interaction patterns, which fully depend on the set of components described in the previous sections.

**Figure 15:**
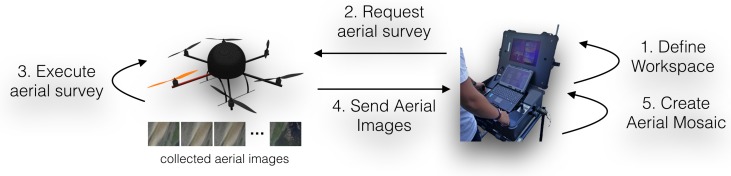
Mission preparation interaction pattern.

Figure 15 depicts the interaction pattern involved in the mission preparation phase, which depends on the elements described in Section 4.2.2 regarding the mosaic creation process. In this phase, the user defines the UAV’s workspace and requests an aerial mosaic, which is performed autonomously by the UAV. At the end of the aerial survey, the UAV sends all acquired images to the base station for subsequent mosaicking. If the resulting aerial mosaic is not adequate, then the whole process is repeated. Aerial mosaic creation fails mostly due to the lack of features in the acquired images, which can result from poor lighting conditions, untextured terrain or inadequate camera poses during acquisition. Although the UAV path is generated automatically (see Section 4.2), the generated way-points can be tuned by the human operator in order to circumvent some of the aforementioned issues.

Interactions between base station and aerial vehicle are ensured by asynchronous ROS messages over the 2.4-GHz wireless network. The high-volume data transmission of all aerial images to the base station may cause undesired network latency; however, as it is confined to a small time window immediately after the aerial survey, the impact is limited.

**Figure 16:**
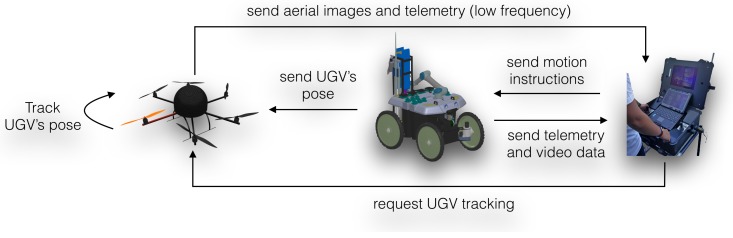
Mission execution interaction pattern.

Figure 16 depicts the main interaction pattern for the mission execution phase. In this interaction pattern, the human expert is assumed to be controlling the UGV from the base station. Alternative interaction patterns may involve having the human operator controlling the robot directly from the RF controller. This may be useful in more critical sampling points. Through the GUI available at the base station, the operator controls the robot by sharing commands and telemetry data with the UGV as asynchronous ROS messages over the 2.4-GHz wireless link. As already mentioned, to avoid undesired latency in key telemetry and coordination messages, video feedback from the UGV’s on-board cameras is provided to the user over a video-dedicated 5.0-GHz wireless link.

Although the on-board cameras provide the operator with a wide perspective of the environment, a third-person perspective is often valuable. A third-person perspective is provided by the aerial vehicle as soon as it is required to track the UGV. The need for tracking the UGV may result from alarms produced by the diagnostic system or due to the need for tracking to be done by following the GPS position of the UGV with a simple PID controller. The UGV pose is cast to the UAV via asynchronous ROS messages over the 2.4-GHz wireless link. The aerial images generated by the UAV are compressed (JPEG) and then transmitted as asynchronous ROS messages to the base station for human visual inspection. The transmission rate is dynamically chosen so as to cope with bandwidth availability.

## 6. Field Trials

This section presents a set of field trials with the purpose of studying: (1) the robustness of the robotic system in terms of mobility and sampling capabilities; (2) the feasibility of the simplified experimental protocol; (3) the logistic issues involved throughout the sampling process; and (4) the set of potential improvements to be taken into account so as to enable a large-scale monitoring campaign. Videos of the field trials can be found on-line (videos from the field trials: https://goo.gl/2tx37k).

### 6.1. Sampling Locations Selection

Inspired by the sampling procedure described in Section 3.3, a small-scale experimental procedure was devised with the specific purpose of assessing the efficiency and robustness of the presented robotic system, in particular of the drilling tool. Concretely, a 1 km-long longitudinal transect along and close to the shore line at the Tagus’ estuary was sampled by the robot at two sites. The two sampling sites along this transect were defined nearby two riverine beaches with road access. The two sites, *A* and *B* hereafter, are ≈1 km apart from each other and were sampled on 29/04/2016 and 19/05/2016, respectively.

**Figure 17:**
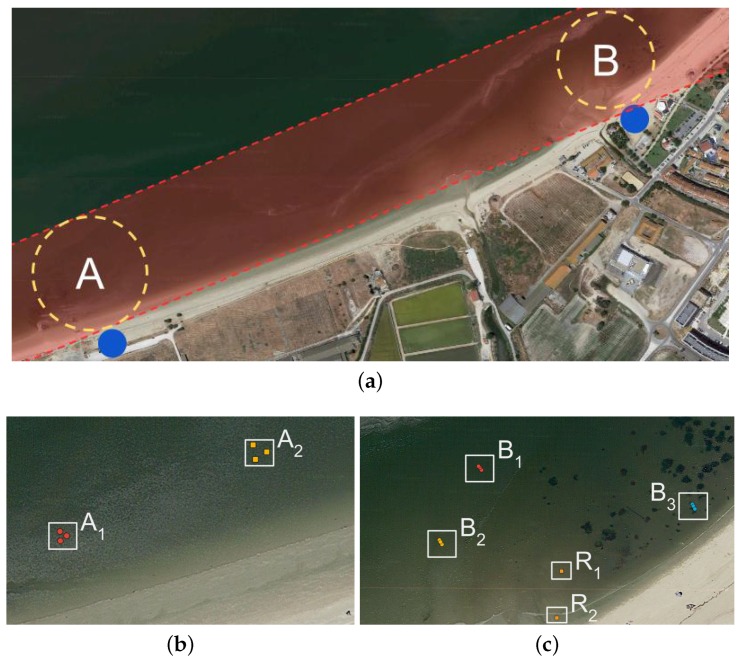
The field trials sites. (**a**) Satellite image covering the field trials sites. The circles represent the two sampling sites, *A* and *B*, visited during the field trials along the longitudinal transect. The filled circles represent the locations of the base station in both sampling sites. (**b**) A close-up perspective over site *A*; (**c**) a close-up perspective over site *B*. The several labels in (b,c) represent sampling locations. The depicted satellite images were acquired during a high tide, which explains why the overlaid sampling sites appear on water regions. Courtesy of Mapdata © 2016 Google.

Figure 17 presents satellite imagery of the selected longitudinal transect with sampling sites overlaid. The depicted satellite images were acquired during a high tide, which explains why the overlaid sampling sites appear on water regions. Conversely, the field trials were performed during the low tide period, allowing the robot to sample the river’s bottom sediments. The actual appearance of the sampling sites during the field trials is depicted in Figure 1.

Rather than selecting randomly the sampling areas, as suggested by the experimental protocol, we have selected areas in a way that would allow us to assess the robot’s drilling execution in different types of terrain. We were particularly interested in checking whether the presence of sea grass, small stones and clams would hamper the drilling process.

In site *A*, two sampling areas were selected, $A_{1}$ and $A_{2}$. Then, three sampling points were randomly selected in each of the two areas. In site *B*, three sampling areas were selected, $B_{1}$, $B_{2}$ and $B_{3}$. However, differently from site *A*, in site *B*, sampling points were not selected randomly. Instead, three evenly-spaced sampling points in a line perpendicular to the river’s shoreline were selected. These points were separated by 1 m. The goal was to test an alternative sampling pattern. Figure 17 presents the location of all sampling points overlaid on satellite imagery.

At each sampling point, the robot collected a bottom sediment sample. When required, filled sample containers were replaced by empty containers in the base station. All sediment samples have been stored by the environment expert in the portable isothermal box and dispatched to the lab for their post-processing.

### 6.2. Logistics

**Figure 18:**
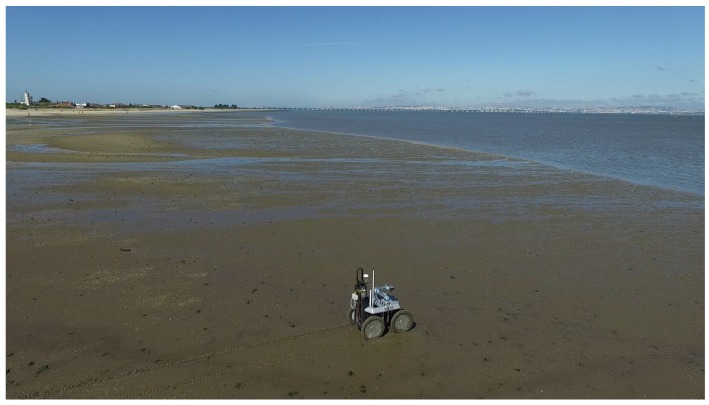
Monitoring mission execution with the aerial vehicle.

Once having arrived at the site, the team sets the base station on a portable table, another portable table for handling the samples, a portable freezer for storing the samples, communications antennas and, finally, the robotic team. Then, it is necessary to build an aerial mosaic of the sampling site before using the ground vehicle for the actual sediment sampling phase. The aerial vehicle can also be used during the mission execution to monitor the ground vehicle’s operations (see Figure 18).

Despite all of these logistic steps, traditional man-based sampling campaigns are even more demanding in this regard. For instance, they often require setting custom hardware to help with localizing the sampling locations. It is also necessary to transport the sample containers and tools. This is particularly critical when the sampling points are far from the base station.

### 6.3. Testing Ground Mobility

Throughout the field trials, the robot traveled ≈5 km. Before engaging in the sampling procedure, the robot was tele-operated with the RF controller from site *A* to site *B*, and then back to *A*. The goal was to test the ability of the robot to move continuously in difficult terrain over an extended period. During this traversal, the robot traveled ≈2.3 km over various kinds of terrain present in the mudflat and spent ≈36% of its battery charge. The battery pack delivered an average of 37 A during this traversal, with a standard deviation of 14 A and a maximum peak of 80 A. These values show that the mobility effort of the robot varied considerably along the course, in particular when crossing the muddy segments.

The locomotion system of the ground vehicle was shown to be able to traverse dry loose sand, muddy terrain and even water-covered terrain. It was ensured that the water level never surpassed the electronic cases’ bottom, that is the maximum water depth allowed was 25 cm. The temperature in the robot’s cases was monitored to check whether any safety threshold would be reached. This test confirmed that the mechanical and electrical design is suited for the task at hand. From all terrain types, the heavily muddied ones were shown to be the most challenging to traverse. In this kind of terrain, walking is practically impossible, even if the expert is carrying no equipment.

### 6.4. Drilling Robustness

**Figure 19:**
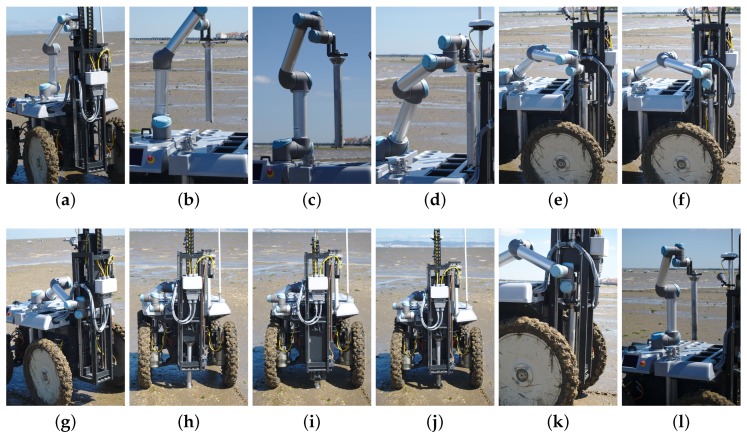
Snapshots of the sampling procedure. (**a**)–(**f**) Robot moving the sampling tube from the storage area to the drilling tool. (**g**)–(**j**) The robot retrieves the core using the drilling tool. (**k**) and (**l**) Robot returning the sampling tube to the storage area.

At each sampling point, the ground vehicle was set to collect a sediment sample. This includes having the robotic arm picking an empty tube from its storage area (see Figure 19a–d). Then, the robotic arm places the empty tube in the drilling tool. A mechanical latch keeps the tube in place (see Figure 19e–g). Then, the tool drills the terrain with the empty tube until the desired depth is reached (see Figure 19h,i). Finally, the filled tube is extracted and stored in the robot’s storage area by the robotic arm (see Figure 19j–l).

**Figure 20:**
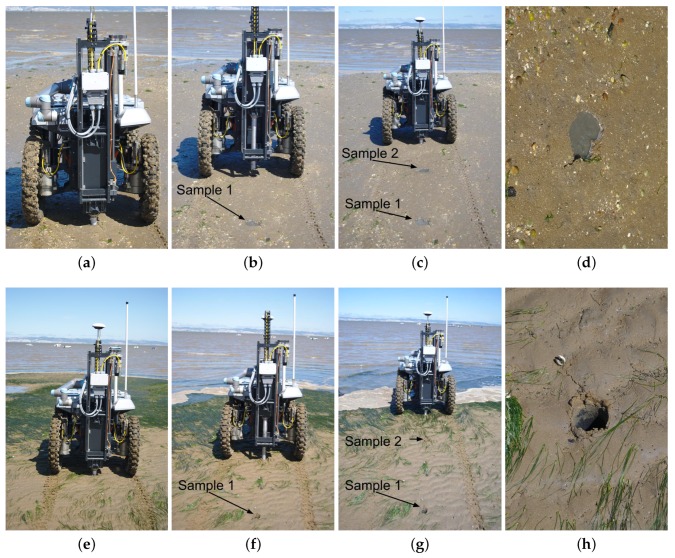
The ground vehicle during two sampling runs. (**a**)–(**d**) Sampling process in location $B_{2}$, (**e**)–(**h**) Sampling process in location $B_{3}$.

Figure 20 depicts the robot performing two sequences of three evenly-spaced sampling locations, roughly separated by 1 m. The two sequences depicted are representative of two types of terrain found during the field trials. The drilling tool was shown to be able to perforate these and all other sampling locations, coping with all terrain types found in the testing environments. Nevertheless, some zones were drilled more easily than others. The muddier the terrain, the less effort had to be made by the drilling tool in the task. This effort variation can be depicted in Table 1, which accounts for both drilling and extraction phases. In some cases, the drilling tool found small solid objects in the soil (e.g., small rocks, shells). In theses cases, the drill kept pushing; the robot raised slightly above the ground; and eventually, the obstacle was overcome, and the drilling process proceeded until the desired depth.

### 6.5. Drilling Performance

Three performance metrics were considered in our study. One refers to the ability of the system to retain the sediment sample in the hollow tube after extraction. Losing sediment means losing information regarding the distribution of contaminants as a function of depth. The second metric refers to the ability of the drilling process to maintain the sample integrity, that is that the sediment is not revolved to the point that depth information is lost. Finally, the third metric is about determining how fast the system is when compared to a human equipped with a manual tool.

**Figure 21:**
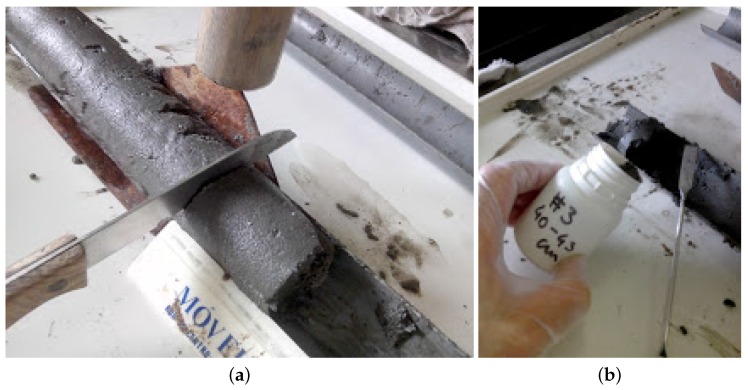
Sample collected by the robotic system being post-processed in the lab. (**a**) The sample in its PVC case; (**b**) profile layers are kept for further processing.

To assess the first metric, a set of 23 collected samples was systematically analyzed (see Figure 21). While 10 of these samples were acquired during the two field trials, the other 13 were drilled in previous testing sessions. On average, the length of the sample is 86% of the PVC tube, with a Standard Deviation (SD) of 14%. In volume and mass terms, this sediment capture efficiency corresponds to average values of 1094.1 cm${}^{3}$ (SD 173.2 cm${}^{3}$) and 1316.3 g dry mass (SD 208.4 g). Sediment sampling performance totally agrees with the mass/volume requirements for off-line analysis. Given the fact that samples are collected in wet soil, the drilling process is shown to be very efficient in avoiding the collected sediment draining out of the tube. As is possible to see from Figure 5e, samples fill a large portion of the PVC tube. Only when drilling under water, which is not an operational requirement, sediments were observed to drain out from the tube.

An empirical observation of the extracted samples also shows that the drilling tool is revolving the sediment only by minimal amounts. Therefore, the samples do not seem to become invalidated by cross-depth contamination. This can be observed from the high density (compression) that the sample in Figure 5e exhibits. At this point, we are only able to assess this metric in qualitative terms. Further larger-scale field trials are required to obtain significant quantitative results.

To assess the efficiency of the drilling process against a human expert, a simple experiment was set. The robot had to perform the whole sample acquisition process, which includes picking an empty tube, drilling and storing the tube in its storage socket. In parallel, a human expert used a typical manual tool to extract a sediment sample (see Figure 2). This human vs. machine race was run in two distinct areas, labeled as $R_{1}$ and $R_{2}$, at sampling site *B* (see Figure 17c). Using traditional techniques, the expert used only 45% and 70% of the time spent by the robot for sampling area $R_{1}$ and $R_{2}$, respectively. However, in area $R_{1}$, the expert’s tools were unable to retain the sample in the tool. The sampling point was covered with a thin water layer. Conversely, the robot managed to sustain most of the sample within the tube.

A closer look at the process unties this apparent tie between man and machine. A large-scale sampling campaign requires performing a large number of drills. The more the samples, the more traverses from the field to the base station are required. Performing these traversals in muddy and sandy terrain while carrying samples and equipment is extremely demanding for humans; not so much for robots. Moreover, the type of soil affects much more the expert than the robot. This is noticeable in the increment of time spent by the expert in $R_{2}$, when compared to $R_{1}$. Differences in terrain composition affect expert’s performance much more than they affect robot’s performance.

The motion speed of the robotic arm and of the drilling tool have been set low purposely, for safety reasons. We believe these could be speeded up to the point of approaching the expert’s performance. However, a detailed analysis and additional field tests are required to confirm this intuition.

## 7. Conclusions

A ground-aerial robotic team capable of bottom sediment sampling in estuarine mudflats was presented. The ground vehicle has been devised for advanced mobility in muddy terrain via a careful weight distribution, materials’ selection and locomotion system design. Sediment sampling is attained via a drilling system that combines rotary and linear motion to reduce mechanical stress and sediment mixing during the sampling process. The sample containers have been designed to avoid dropping the samples during transport to the base station. Sampling procedures are highly complex to design and, so, returning to human experts is essential. Bearing this in mind, an aerial vehicle has been added to the team. This vehicle gathers aerial imagery for the expert to plan and monitor mission execution. The robot’s control systems rely on ROS, which has been a valuable tool for fast prototyping and integration of modules previously developed for other robotic systems. It also enables an interesting framework for the diagnostic system, which is key in field robots performing long-lasting operation. Towards closing the gap between robotics and environmental monitoring, we propose a preliminary experimental protocol for the specific case of routine radiological and heavy metals monitoring in estuarine mudflats, given the characteristics of the robotic system. In fact, both the robots and the experimental protocol were co-developed according to the mutual constraints that have been found throughout the process. This multidisciplinary experiment shows how relevant it is to involve the environment scientists early in the design process. A set of field trials showed promising results in terms of the robotic system’s robustness. More importantly, they showed that the collected samples are proper for lab post-processing.

We are currently preparing a full environmental monitoring campaign to validate the proposed experimental protocol and further assess the robustness and accuracy of the robotic system when facing the burdens of long field operations. As future work, we expect to allow the expert to dynamically (re)design the mission workflow, i.e., the set of interactions between operator and robotic team mates [50]. In the present study, the ground vehicle has been tele-operated to fulfil its mission. In the future, we intend to study the impact of autonomous behavior in the mission workflow. Automatic recharging of the sample containers once these are filled would help to reduce the physical engagement of the human expert. Additionally, it would be greatly advantageous if the aerial mosaic could be automatically segmented into the key features of the environment (e.g., water ponds, sea grass coverage). Ideally, the system should be able to progressively refine its image classifiers from data gathered in previous missions. We also expect to include energy-aware multi-robot path planning strategies so as to enable efficient soil sediment sampling campaigns in demanding environments (e.g., non-planar, with large areas of loose sand). In fact, long-lasting operation in remote sites may also require the path planner to also take into account energy harvesting activities. A self-washing mechanism will be included in the robotic solution in order to avoid material wearing and sensors/actuators malfunction. In addition, the robotic system will be extended so as to include our water surface unmanned vehicle [16] for joint water/land sampling. The experimental protocol will also be adapted to account for the extended robotic team. Finally, we also intend to fit our previous work in cooperative terrain perception of aerial-aquatic robotic teams [16] to the aerial-ground case. The goal is to to expand the ground vehicle’s look-ahead capabilities, fostering more efficient and safer navigation in demanding terrain.

## Figures and Tables

**Table 1:** Average ± standard deviation of the current delivered to both the linear and rotation actuators of the sampling tool, over three sampling points. Each line of the table corresponds to a sampling area. All values are in amperes and have been obtained separately for the drilling and the extraction phases.

	Drilling Phase		Extraction Phase	
Area	Linear Actuator	Rotation Actuator	Linear Actuator	Rotation Actuator
$A_{1}$	2.94±1.20	3.87±7.33	3.12±0.83	N/A
$A_{2}$	2.63±0.49	2.80±4.27	2.84±0.83	N/A
$B_{1}$	3.10±0.80	1.23±1.76	2.68±1.00	N/A
$B_{2}$	4.09±0.88	3.71±6.89	3.09±1.03	N/A
$B_{3}$	4.26±1.09	2.84±5.02	3.05±(0.88)	N/A

## Supplementary Materials

Videos of the robotic system during the field trials can be found at https://goo.gl/2tx37k.

## Abbreviations

GUI	Graphical User Interface
UAV	Unmanned Aerial Vehicle
UGV	Unmanned Ground Vehicle
GPS	Global Positioning System

## References

[1] Eggleton J., Thomas K.V. (2004). A review of factors affecting the release and bioavailability of contaminants during sediment disturbance events. Environ. Int..

[2] Guedes M., Santana P., Deusdado P., Mendonça R., Marques F., Henriques N., Lourenço A., Correia L., Barata J., Flores L. ARES-III: A versatile multi-purpose all-terrain robot. Proceedings of the 2012 IEEE 17th Conference on Emerging Technologies & Factory Automation (ETFA).

[3] ICRU (2006). Sampling to Estimate Spatial Pattern.

[4] Deusdado P., Pinto E., Guedes M., Marques F., Rodrigues P., Lourenço A., Mendonça R., Silva A., Santana P., Corisco J. (2016). An aerial-ground robotic team for systematic soil and biota sampling in Estuarine Mudflats. Robot 2015: Second Iberian Robotics Conference.

[5] Smith L.C. (1997). Satellite remote sensing of river inundation area, stage, and discharge: A review. Hydrol. Process..

[6] Mainwaring A., Culler D., Polastre J., Szewczyk R., Anderson J. (2002). Wireless sensor networks for habitat monitoring. Proceedings of the 1st ACM International Workshop on Wireless Sensor Networks and Applications.

[7] Rundel P.W., Graham E.A., Allen M.F., Fisher J.C., Harmon T.C. (2009). Environmental sensor networks in ecological research. New Phytol..

[8] Song W.Z., Huang R., Xu M., Ma A., Shirazi B., LaHusen R. (2009). Air-dropped sensor network for real-time high-fidelity volcano monitoring. Proceedings of the 7th International Conference on Mobile Systems, Applications, and Services.

[9] Capella J.V., Bonastre A., Ors R., Peris M. (2014). A step forward in the in-line river monitoring of nitrate by means of a wireless sensor network. Sens. Actuators B Chem..

[10] Li M., Liu Y. (2009). Underground coal mine monitoring with wireless sensor networks. ACM Trans. Sens. Netw..

[11] Bhadauria D., Isler V., Studenski A., Tokekar P. A robotic sensor network for monitoring carp in Minnesota lakes. Proceedings of the 2010 IEEE International Conference on Robotics and Automation (ICRA).

[12] Bajracharya M., Maimone M.W., Helmick D. (2008). Autonomy for mars rovers: Past, present, and future. Computer.

[13] Thrun S., Thayer S., Whittaker W., Baker C., Burgard W., Ferguson D., Hähnel D., Montemerlo M., Morris A., Omohundro Z. (2004). Autonomous exploration and mapping of abandoned mines. IEEE Robot. Autom. Mag..

[14] Kantor G., Fairfield N., Jonak D., Wettergreen D. (2008). Experiments in navigation and mapping with a hovering AUV. Field and Service Robotics.

[15] Kimball P., Bailey J., Das S., Geyer R., Harrison T., Kunz C., Manganini K., Mankoff K., Samuelson K., Sayre-McCord T. The whoi jetyak: An autonomous surface vehicle for oceanographic research in shallow or dangerous waters. Proceedings of the IEEE/OES Autonomous Underwater Vehicles Conference.

[16] Pinto E., Marques F., Mendonça R., Lourenço A., Santana P., Barata J. An autonomous surface-aerial marsupial robotic team for riverine environmental monitoring: Benefiting from coordinated aerial, underwater, and surface level perception. Proceedings of the IEEE International Conference on Robotics and Biomimetics (ROBIO).

[17] Murphy R.R., Peschel J., Arnett C., Martin D. Projected needs for robot-assisted chemical, biological, radiological, or nuclear (CBRN) incidents. Proceedings of the IEEE International Symposium on Safety, Security, and Rescue Robotics (SSRR).

[18] Dunbabin M., Marques L. (2012). Robots for environmental monitoring: Significant advancements and applications. IEEE Robot. Autom. Mag..

[19] Marques F., Lourenço A., Mendonça R., Pinto E., Rodrigues P., Santana P., Barata J. A critical survey on marsupial robotic teams for environmental monitoring of water bodies. Proceedings of the IEEE OCEANS.

[20] Wilkins J.W. (1977). Drilling Sampling/Testing Equipment. U.S. Patent.

[21] Philipenko H. (1982). Soil Sampler and Mounting Arrangement. U.S. Patent.

[22] Doty J.G. (1982). Bumper Mounted Soil Sampling Device. U.S. Patent.

[23] Sneath R., Phillips V., Price J. (1989). Powered soil samplers for heavy metals and some concepts for the future. J. Agric. Eng. Res..

[24] Nosewicz M.A., Turner S.B. (1993). Portable Soil Sampling Device and Method. U.S. Patent.

[25] Wright N.A., Wright H.L. (1999). Extended Soil Sampling Head. U.S. Patent.

[26] Naber R.J., Naber G.G. (2002). Soil Sampling Device. U.S. Patent.

[27] Porritt J., Scott H. (1991). Vibratory Core Drill Apparatus for the Recovery of Soil or Sediment Core Samples. U.S. Patent.

[28] Marker R. (2013). Vehicle Mounted Soil Sampler. U.S. Patent.

[29] Bacchelli A., Catone G. (2011). A Core Sampling Apparatus. EP Patent App..

[30] Wright N.A., Wright H.L. (1995). Mobile Soil Sampling Device. U.S. Patent.

[31] Pavlik J. (2009). Mobile Soil Sampling Device With Vacuum Collector. U.S. Patent.

[32] Edwards R.D., Smith A.E. (1989). Automatic Soil Sampling Machine. U.S. Patent.

[33] Hale G. (2000). Soil Sampling “on the Fly”. U.S. Patent.

[34] Dagel J.H., Hesse M.B., Ackerman R.J. (2004). Rotary Soil Probe. U.S. Patent.

[35] Burton J.D. (2010). Soil Sampling Apparatus and Method. U.S. Patent.

[36] Guzman R., Navarro R., Ferre J., Moreno M. (2015). RESCUER: Development of a modular chemical, biological, radiological, and nuclear robot for intervention, sampling, and situation awareness. J. Field Robot..

[37] Winter A.G., Deits R.L., Dorsch D.S., Hosoi A.E., Slocum A.H. Teaching roboclam to dig: The design, testing, and genetic algorithm optimization of a biomimetic robot. Proceedings of the IEEE/RSJ International Conference on Intelligent Robots and Systems (IROS).

[38] Darukhanavala C., Lycas A., Mittal A., Suresh A. Design of a bimodal self-burying robot. Proceedings of the IEEE International Conference on Robotics and Automation (ICRA).

[39] Carvalho F.P., Oliveira J.M., Silva L., Malta M. (2013). Radioactivity of anthropogenic origin in the Tejo Estuary and need for improved waste management and environmental monitoring. Int. J. Environ. Stud..

[40] Caetano M., Madureira M.J., Vale C. (2003). Metal remobilization during resuspension of anoxic contaminated sediment: Short-term laboratory study. Water Air Soil Pollut..

[41] Santana P., Cândido C., Santos P., Almeida L., Correia L., Barata J. (2008). The Ares robot: Case study of an affordable service robot. Proceedings of the European Robotics Symposium (EUROS), Diplomat Hotel Prague.

[42] Marques F., Santana P., Guedes M., Pinto E., Lourenço A., Barata J. Online self-reconfigurable robot navigation in heterogeneous environments. Proceedings of the IEEE International Symposium on Industrial Electronics (ISIE).

[43] Quigley M., Gerkey B., Conley K., Faust J., Foote T., Leibs J., Berger E., Wheeler R., Ng A. ROS: An open-source robot operating system. Proceedings of the ICRA Open-Source Software Workshop.

[44] Mace J. Rosbridge. http://wiki.ros.org/rosbridge_suite.

[45] Santana P.F., Cândido C., Santos V., Barata J. A motion controller for compliant four-wheel-steering robots. Proceedings of the IEEE International Conference on Robotics and Biomimetics (ROBIO).

[46] Sucan I.A., Chitta S. MoveIt!. http://moveit.ros.org/.

[47] Arducopter-Open-Source Multicopter Controller. http://www.arducopter.co.uk/.

[48] Photo Stitcher H.P. D’Angelo, Pablo. http://hugin.sourceforge.net.

[49] Axis Communications AB (2013). VAPIX Version 3 Video Streaming API. http://www.axis.com/files/manuals/vapix_video_streaming_52937_en_1307.pdf.

[50] Santana P., Correia L., Salgueiro M., Santos V., Barata J. A Knowledge-based component for human-robot teamwork. Proceedings of the International Conference on Informatics in Control, Automation and Robotics (ICINCO).

